# Supplementary material for: Integrated multi-omics analysis reveals necroptosis-related biomarker BIRC3 for early diagnosis and therapeutic targeting in preeclampsia
Source: PLoS One. 2025 Dec 1;20(12):e0337546. doi: 10.1371/journal.pone.0337546 (PMC12668491; doi:10.1371/journal.pone.0337546)
Supplement: Attachment — Submitted filename: 3.Response to Reviewers.docx [file pone.0337546.s006.docx]

### ****Response to Reviewers****

**Manuscript ID:** PONE-D-25-39907
**Title:** Integrated multi-omics analysis reveals necroptosis-related biomarker BIRC3 for early diagnosis and therapeutic targeting in preeclampsia

We sincerely thank the editors and reviewers for their time, insightful comments, and constructive suggestions, which have significantly helped us improve the quality of our manuscript. We have carefully considered all points raised and have revised the manuscript accordingly. Our point-by-point responses are detailed below. All changes in the manuscript have been highlighted in the "Revised Manuscript with Track Changes" file.

#### ****Response to Editor:****

1. **Style Requirements:** We have carefully reviewed and formatted our manuscript to ensure it fully complies with PLOS ONE's style requirements, including file naming conventions.
2. **Code Sharing:** The analyses in this study were performed using standard R packages and publicly available software tools (Xiantao platform and Home for researchers platform). No custom code was generated that underpins the central findings. We also extend our gratitude to these public analysis platforms in the acknowledgments section **(Page 27, line 542-546)**, appreciating the efficient and reliable data analysis environment they provided for our research.
3. **ORCID iD:** We confirm that the corresponding author's ORCID ID has been validated in Editorial Manager. (Guili Shi: 0009-0002-4263-4872; Yanfeng Lu: 0009-0004-2638-2198).
4. **Copyright for Figure 1:** We thank the editor for this important reminder. We have replaced the original Figure 1 with a new, self-created figure that does not contain any copyrighted material. The new figure is entirely original and is prepared by the authors for this publication, thus it complies fully with the CC BY 4.0 license.
5. **Copy of Table 1:** We thank the editor for pointing this out. As requested, a copy of Table 1 has now been included in the main manuscript file on **Page 15.**
6. **Supporting Information Captions:** Captions for all supporting information files have been included at the end of the manuscript (**Page 39**).

#### ****Response to Reviewer #1:****

We are grateful to Reviewer #1 for the thorough and constructive feedback, which has greatly strengthened our manuscript. We have addressed each point in detail below.

**Comment 1: Introduction could be improved with a more focused research gap, better justification for bioinformatics, and improved citation alignment.**

**Response:** We sincerely thank the reviewer for these constructive suggestions regarding the Introduction. We have thoroughly revised this section to address the raised concerns:

(1) Critical Appraisal of Prior Work and Articulation of the Research Gap:

We agree that our initial statement regarding the research gap was too broad. In the revised Introduction, we have provided a more critical and specific appraisal of the existing literature on necroptosis in PE. We now cite and discuss key foundational studies that have begun to implicate necroptosis in PE **(Page 3-6).**

(2) Strengthened Justification for Bioinformatics Approach:

The transition to bioinformatics has been enhanced with a clear rationale. We now explicitly state that integrated bioinformatics serves as a powerful strategy for hypothesis generation from public genomic data, enabling systematic identification of high-confidence candidates and construction of coherent biological narratives to guide future research **(Page 5-6，line 102-106)**.

(3) Reframed the Final Paragraph with a Clear Hypothesis and Objectives:

We have completely rewritten the final paragraph to replace the previous simple listing of methods. It now clearly states our main hypothesis, that specific necroptosis-related genes are dysregulated in PE and contribute to its pathogenesis. Additionally, it outlines our specific research objectives, which are designed to test this hypothesis. This revision has created a much stronger and more focused narrative arc **(Page 5-6)**.

(4) Improved Citation Alignment and Literature Review:

We have updated the Introduction to include key references that are also discussed later in the manuscript (References 10-21), ensuring better flow and eliminating the citation gap. The literature review now more accurately reflects the current state of the field regarding cell death pathways in PE.

**Comment 2: DEG screening criteria (p<0.05 without multiple testing correction) risk false positives. Preprocessing details are insufficient.**

**Response：**We sincerely thank the reviewer for raising this critical point regarding multiple testing correction. We fully agree that controlling the false discovery rate (FDR) is a cornerstone of rigorous high-throughput data analysis.

In designing our analysis, we carefully considered the balance between statistical stringency and the risk of false negatives (Type II errors). Given that our primary dataset (GSE66273) has a relatively **small sample size (n=11)**, applying a strict FDR correction (e.g., BH method) can be overly conservative and might filter out biologically relevant genes with moderate but consistent changes in expression, which are common in pilot or exploratory studies like ours. Therefore, we opted to use an unadjusted **p-value < 0.05** combined with a **|log_2_FC| > 1** threshold as our initial screening criteria. We acknowledge in our revised manuscript that this approach carries a higher risk of false positives compared to FDR correction.

**However, to mitigate this risk and increase confidence in our findings, we did not rely solely on this initial p-value screening. The core gene, BIRC3, was subsequently validated through a multi-layered, independent analytical framework:**

（1）**Protein-Protein Interaction (PPI) Network:** BIRC3 was identified as a hub gene based on its network topological properties, which is independent of its p-value.
（2）**Machine Learning (Random Forest):** BIRC3 was prioritized by the Random Forest algorithm due to its importance in classifying PE vs. control, providing a separate, model-based validation of its relevance.
（3）**Immune Infiltration Correlation:** The expression of BIRC3 showed significant correlations with specific immune cell populations, adding biological context to its role.
（4）**Single-cell RNA-seq Validation:** The specific expression of BIRC3 in key placental immune cells (e.g., Hofbauer cells, T cells) from an **independent dataset (GSE173193)** provides strong orthogonal evidence supporting its biological significance in PE.

The convergence of evidence from these diverse and independent methods suggests that BIRC3 is a robust finding. We have now explicitly stated this consideration and the inherent limitation in the study Limitations section **(Page 25, line 504-508)**. We believe that this validation strategy effectively compensates for the less stringent initial filter and reinforces the validity of our central conclusion regarding BIRC3.

**Comment 3:** The selection of necroptosis-related genes from GeneCards requires clearer filtering criteria.

**Response：**We thank the reviewer for this valuable suggestion to clarify our methodology. In response, we have revised the manuscript to provide a detailed description of the filtering criteria used for selecting necroptosis-related genes from the GeneCards database.

Specifically, the initial gene list for "necroptosis" was retrieved from the GeneCards database. To ensure we focused on genes with a strong and specific association to necroptosis, we applied a stringent threshold. Specifically, we calculated the median relevance score of all genes associated with the term "necroptosis" in GeneCards and retained only those genes with a relevance score greater than or equal to twice the median value.

Crucially, we have re-performed all subsequent analyses based on this updated list of necroptosis-related DEGs. This includes regenerating **Figure 4** and re-conducting the associated functional enrichment analysis. We are pleased to confirm that this more stringent gene selection, while refining the specific inputs, did not alter our central finding: BIRC3 remained a key necroptosis-related gene emerging from the analysis, and the overall conclusions of the study remain fully supported.

This refined list of high-confidence necroptosis-related genes (**S1 table1**) was then used for the subsequent intersection with our DEGs . We have updated the 'Analysis of DEGs' subsection in the Methods **(Page 7, line 136-139)** to include these details.

**Comment 4: while the authors correctly note that public datasets do not require new ethics approval, it would be appropriate to clarify that the original dataset had obtained ethical clearance.**

**Response：We thank the reviewer for this appropriate suggestion. We have now clarified the ethical compliance of the original studies in the Data Acquisition subsection of the Methods section. The revised text states: All datasets analyzed in this study were obtained from the public Gene Expression Omnibus (GEO) database. The original studies associated with these datasets (GSE66273, GSE173193, GSE44711) had received necessary ethical approval, as documented in their respective source publications** (**Page 28, line 557-560**) **.**

**Comment 5: scRNA-seq workflow description is incomplete (QC, PC selection, annotation validation).**

**Response：**We thank the reviewer for this insightful comment. We agree that a comprehensive description of the scRNA-seq analysis workflow is essential for reproducibility. In response, we have thoroughly revised the 'Single-cell RNA sequencing (scRNA-seq) analysis' subsection in the Methods **(Page 8-9, line 151-169)** to include the missing details. We believe that these additions have enhanced the clarity, rigor, and reproducibility of our scRNA-seq analysis.

**Comment 6: Immune infiltration analysis with xCell may be limited for placental tissue, and no sensitivity analyses are described. The rationale for using both ssGSEA and xCell is unclear.**

**Response:** We sincerely thank the reviewer for these insightful comments, which have helped us significantly improve the rigor of our immune infiltration analysis.

(1) Rationale for using both ssGSEA and xCell: We acknowledge that the rationale for employing two algorithms was not clearly stated in the original manuscript. We have now clarified this in the revised Methods section **(Page 9, line 171-185)**. Our intent was to leverage the complementary strengths of different computational approaches to triangulate our findings and enhance confidence in the observed immune patterns. Specifically: ssGSEA evaluates the enrichment of predefined gene signatures for specific cell types, providing a pathway-centric view. xCell employs a novel gene signature-based method that can deconvolve a broader spectrum of cell types (64 immune and stromal cells) and is considered to have improved accuracy for closely related cell types. While we acknowledge some inherent variability between algorithms when applied to placental tissue, this analysis specifically demonstrates that a consistent trend of immune dysregulation in PE is observed across all two methods.
(2) We performed a sensitivity analysis using CIBERSORTx (**Figure 1 in this document**). The results revealed that monocytes were the only group showing significant differences. In contrast, there were no statistically significant differences in the infiltration levels of various cells, including macrophages M0, M1 and M2, among the groups. We acknowledge that the results from CIBERSORTx were not entirely consistent with those from ssGSEA and xCell regarding the specific subsets and magnitudes of immune cell changes. We interpret these divergent results as highlighting a crucial methodological limitation: computational deconvolution tools, which are largely trained on and optimized for blood or immune cell contexts, exhibit significant variability and inherent uncertainty when applied to the complex and unique tissue microenvironment of the human placenta. We have added a statement in the Discussion regarding the potential limitation of deconvolution algorithms for placental tissue **(Page 25, line 508-511)**. Therefore, while we present the immune deconvolution results as an exploratory analysis suggesting a potential role for immune dysregulation, we have deliberately refrained from building our core conclusions on these specific immune cell estimates.

**Figure 1. Analysis of immune cell infiltration using CIBERSORTx.** (A) Box plot of immune cell proportions between the Normal and PE group. **P < 0.01. (B) Heatmap of the immune cell infiltrates based on the CIBERSORTx.

(3) Strengthening the study's foundation beyond immune deconvolution: We wish to emphasize that the central narrative of our manuscript—the identification and validation of BIRC3 as a key necroptosis-related gene in PE—does not rely on the immune deconvolution results. The role of BIRC3 is robustly supported by independent and more definitive lines of evidence, including: ① Its identification as a hub gene in the PPI network. ② Its high feature importance in the machine learning (Random Forest) model. ③ Most importantly, our single-cell RNA-seq analysis provides direct and independent evidence that strengthens our key findings from two perspectives: First, it precisely localizes the expression of the key necroptosis-related gene BIRC3. We found that BIRC3 is not restricted to a single cell type but is predominantly expressed across multiple immune cell populations in the placenta, including T cells, B cells, and Hofbauer cells. Second, this single-cell data independently confirmed a broad dysregulation of the immune microenvironment in PE. The analysis revealed significant alterations in the proportions of various immune and other cell types between PE and control placentas.

Therefore, we have refined our interpretation in the manuscript. The combined evidence leads us to a more nuanced and robust conclusion: the core issue in PE is a generalized imbalance and dysfunction of the placental immune microenvironment, rather than a change in one specific, precisely quantifiable immune cell type.

In summary, we have (1) clarified the methodological rationale, (2) transparently addressed the algorithmic inconsistencies, and (3) leveraged scRNA-seq data to independently validate both the expression pattern of BIRC3 and the overarching theme of immune microenvironment dysregulation, thereby strengthening the foundation of our conclusions.

**Comment 7: PPI network confidence threshold is unspecified. Hub gene identification relies solely on topology.**

**Response: We thank the reviewer for these pertinent observations, which have helped us improve the transparency and robustness of our network analysis.**

(1) **PPI Network Confidence Threshold: We apologize for this oversight. The PPI network was indeed constructed using the STRING database with a minimum interaction score of 0.4, indicating a medium confidence threshold. This specific criterion has now been clearly stated in the revised Methods section (Page 10, line 192-194).**
(2) Hub Gene Identification and Validation: We acknowledge that the initial identification of candidate hub genes from the PPI network was based on network topology algorithms (degree, EPC, etc.). We have revised the relevant text in the Results section **(Page 15, line 303-305)** to clarify that these were preliminary candidate hubs selected by topological features.

However, we wish to emphasize that the final selection of our core gene, BIRC3, was not reliant solely on the PPI network. Its central role was further corroborated by an independent machine learning approach—the Random Forest algorithm. The fact that BIRC3 was prioritized as a key feature by both network topology and a separate classification model significantly strengthens its candidacy beyond what topological analysis alone could provide.

We have tempered our language in the manuscript to present the PPI-based hubs as a first step in a multi-step filtering process, with the convergence of evidence from different methodologies lending greater confidence to the ultimate selection of BIRC3 **(Page 15-16, line 300-311).**

**Comment 8: ROC analysis lacks external validation, risking overfitting.**

**Response:** **We thank the reviewer for these important comments regarding the need for external validation and the interpretation of our computational predictions. We have addressed these concerns as follows:**

(1) **External Validation of BIRC3 Expression (Figure 2 in this document):**

**We performed an external validation using the independent GEO dataset GSE44711. The analysis yielded two key confirmatory results: ① Consistent Dysregulation: BIRC3 expression was significantly upregulated in preeclampsia placentas compared to normal controls in this independent validation set. ② Sustained Diagnostic Performance: The ROC analysis on this external cohort demonstrated that BIRC3 retains very good diagnostic potential, with an AUC of 0.844 (Page 16, line 312-321).**

(2) **Refinement of Claims in the Manuscript:**

**The language in the Abstract (Page 2-3, line 41-43), Results (Page 16, line 312-321) and Discussion section (Page 23, line 468-473) has been refined to more accurately reflect the strengthened, validated status of BIRC3's diagnostic potential, moving from "preliminary" to "independently validated" while still acknowledging the need for future prospective clinical studies.**

**Figure 2. The expression level and ROC curves of BIRC3.** The first line is the traning set data, the second line is the validation set data. *P < 0.05.

**Comment 9: The TF–mRNA, miRNA–mRNA, and RBP–mRNA networks were constructed purely from prediction databases, with no reported filtering criteria, confidence thresholds, or experimental validation, making their biological significance uncertain.**

**Response:** We thank the reviewer for this critical comment regarding the construction and interpretation of the regulatory networks. We have now revised our methodology description to address these concerns directly.

(1) Clarification of the Analysis Platform and Its Parameters:

The TF–mRNA and miRNA–mRNA networks were constructed using NetworkAnalyst (*Nucleic Acids Res 2019, 47 (W1), W234, doi：10.1093/nar/gkz240； Nat Protoc 2015, 10 (6), 823, doi：10.1038/nprot.2015.052.*), a widely recognized and extensively cited online platform for gene expression and network analysis, which has been validated in numerous peer-reviewed publications. In our revised Method section **(Page 10, line 204-208 and line 212-215)**, we have now provided the specific computational parameters and filtering criteria used within this platform: we specified that only interactions meeting the stringent thresholds of peak intensity signal < 500 and a regulatory potential score < 1 were included, and with the minimal network connectivity.

We constructed the RBP-mRNA interaction network using data from the ENCORI starBase platform (https://rnasysu.com/encori/index.php). The network depicts interactions supported by CLIP-seq-derived RBP binding sites. All identified binding sites within 200bp of a RBP will be defined as a RBP-binding cluster **(Page 11, line 222-224).**

(2) Reframed Presentation as Hypothesis-Generating:

In line with the reviewer's valid point about biological significance, we have reframed the presentation of these networks in the Results and Discussion sections. They are now explicitly described as "high-confidence in silico predictions" that serve a hypothesis-generating purpose **(Page 24, line 476-477)**. We clearly state that their value lies in providing a prioritized, computationally-derived framework to guide future experimental validation (e.g., via ChIP-qPCR or luciferase reporter assays), not as definitive proof of regulatory relationships.

**Comment 10: The drug prediction based on DGIdb is exploratory and lacks pharmacological plausibility or validation. The docking analysis is described superficially, with incomplete reporting of grid parameters, scoring criteria, and validation steps, and it focuses on a single target-ligand pair, which provides only preliminary and hypothetical insights.**

**Response: We thank the reviewer for these critical comments regarding the drug prediction and molecular docking analyses. We agree that these components are exploratory in nature and have revised the manuscript to better reflect this, while also providing the missing methodological details to enhance reproducibility and scientific rigor.**

(1) **Nature of the Drug Prediction and Docking Analyses: We fully acknowledge that the drug prediction and molecular docking results are exploratory and hypothesis-generating. The primary goal of this section was not to propose clinically ready therapeutics, but to demonstrate a computational workflow that bridges biomarker discovery (BIRC3) with potential therapeutic strategies, thereby providing a direction for future experimental research.**
(2) **Enhanced Description of Molecular Docking Methodology: We have thoroughly revised the Methods section (Page 12, line 231-245) to provide a complete and transparent account of the molecular docking procedures.**
(3) **Appropriate Framing and Limitations: We have reframed the presentation of these results throughout the manuscript:**

- **In the Results, we now present the DGIdb-based prediction as an initial screening step to identify candidate compounds with documented biological activities relevant to PE pathophysiology. We explicitly state that the molecular docking results, while showing favorable binding affinities, do not confirm biological activity or therapeutic efficacy (Page 21, line 422-423 and line 429-432).**
- **In the Discussion, we emphasize that these findings are preliminary and that the safety, specificity, and efficacy of these compounds in the context of pregnancy remain entirely unknown and require rigorous experimental validation (Page 24-25, line 484-503).**

**Comment 11: Functional enrichment based on 6 genes is unstable. The complex workflow lacks detail, and Random Forest on a small dataset risks overfitting.**

**Response:** We sincerely thank the reviewer for these insightful comments. We fully acknowledge that functional enrichment analysis based on only six necroptosis-related DEGs (NRDEGs) can **yield unstable results. To address this, we have reframed the presentation of these results in the Results section (Page 14, line 283-293). We now describe this analysis as a preliminary characterization of the biological themes associated with this specific gene set, rather than as a definitive pathway analysis. We emphasize that the primary purpose was to contextualize the six NRDEGs, noting that several (including BIRC3) were implicated in apoptosis and NF-kB signaling, which aligns with known PE pathophysiology. This cautious interpretation is now explicitly stated.**

**Comment 12: The workflow to define hub genes is complex and lacks detail.**

**Response: We thank the reviewer for this comment. We have now expanded the description of our multi-step hub gene identification workflow in the Results section (Page 15, line 300-301) to enhance clarity and reproducibility. The specific procedure is as follows:**

(1) **Protein-Protein Interaction (PPI) Network Construction and Hub Gene Screening: The upregulated differentially expressed genes (DEGs) were used to construct a PPI network using the STRING database, with a minimum confidence score of 0.4. The resulting network was imported into Cytoscape, and hub genes were preliminarily identified using the CytoHubba plugin by applying four topological algorithms: Degree, Edge Percolated Component (EPC), Stress, and Eccentricity.**
(2) **Machine Learning-Based Feature Prioritization: The model was run with default parameters, and genes were ranked by their importance based on the Mean Decrease Gini index. The top 20 feature genes from this analysis were retained for subsequent integration.**
(3) **Integration and Final Hub Gene Selection: To identify the most robust and biologically relevant candidate, we took the intersection of three gene sets:**

- **The 9 candidate hub genes from the PPI network**
- **The 20 top feature genes from the Random Forest model**
- **The 3 necroptosis-related DEGs (NRDEGs)**

**This integrative approach identified BIRC3 as the final hub gene, as it was the only gene common to all three sets.**

**This multi-method strategy was designed to leverage both network topology and machine learning-based feature importance, ensuring the selected hub gene was not only central in the interactome but also critical for sample classification. The convergence of evidence from these independent methods strengthens the confidence in BIRC3 as a key regulator. We have updated the Methods and Results sections to include these details (Page 15-16, line 300-311).**

**Comment 13: Random Forest on a small dataset risks overfitting.**

**Response: The primary goal of employing the Random Forest algorithm in our study was not to build a generalized predictive model, but to leverage it as a robust method for internal feature ranking and prioritization within our discovery cohort. This application is valid for identifying features with high relative importance, even in smaller datasets.**

**Crucially, the prominence of BIRC3 is not reliant on a single analysis. Its selection was independently corroborated by its concurrent identification as a hub gene in the PPI network, an orthogonal methodology. Most importantly, the successful external validation in the independent GSE44711 cohort—where BIRC3 showed consistent dysregulation and strong diagnostic power (AUC = 0.844)—provides definitive evidence that its signal is genuine and generalizable. This multi-layered confirmation, combining internal convergence with external validation, robustly demonstrates that the identification of BIRC3 is a biologically relevant finding and not an artifact of overfitting in the discovery cohort.**

**Comment 14: The claim of being the "first comprehensive multi-omics study" is too strong. Limitations are underdeveloped.**

**Response:** We thank the reviewer for this important comment. We agree that the phrasing was overly strong and that a more detailed discussion of limitations strengthens a scientific manuscript. We have addressed these points as follows:

(1) We have removed the phrase "the first comprehensive multi-omics study" from the Abstract and Discussion. Instead, we now describe our work as "an integrated multi-omics analysis" that "provides a comprehensive resource" for understanding necroptosis in PE **(Page 22, line 442)**. This more accurate phrasing acknowledges our study's integrative approach without making claims of absolute priority that are difficult to substantiate.
(2) We have significantly expanded the Limitations section in the Discussion to provide a more critical and nuanced reflection of our study's constraints **(Page 25, line 504-521).**

#### ****Response to Reviewer #2:****

We thank Reviewer #2 for their positive assessment of our work and their valuable suggestions for improvement.

**Comment 1: The bulk transcriptomic dataset (GSE66273) includes only 6 PE vs. 5 controls, which substantially limits statistical robustness. This limitation should be acknowledged more explicitly in the Discussion. The generalizability of findings should be presented cautiously. Validation in larger independent cohorts (if available) is strongly recommended, or at least a justification for using this dataset should be provided.**

**Response:** We sincerely thank the reviewer for raising this critical point. We fully acknowledge that the limited sample size of the primary discovery cohort (GSE66273, n=11) is a significant constraint. We have addressed this concern through the following approaches, which are now clearly articulated in the revised manuscript:

(1) External Validation in an Independent Cohort: Most importantly, to directly assess the generalizability of our findings, we validated our core result in an independent, publicly available dataset, GSE44711. We are pleased to report that BIRC3 expression was consistently and significantly upregulated in preeclamptic placentas in this external cohort. Furthermore, it demonstrated strong and reproducible diagnostic performance with an AUC of 0.844 (**Figure 2**).
(2) Convergent Evidence from Multiple Methodologies: The significance of BIRC3 is not reliant on a single analysis of the small dataset. It was independently prioritized through a multi-step analytical framework, including protein-protein interaction network topology and machine learning-based feature ranking within the discovery set. The convergence of evidence from these independent methods, followed by successful external validation, strongly supports that BIRC3 is a biologically relevant finding and not an artifact of the initial sample size.

**Comment 2: Use GeneCards for necroptosis genes; a curated pathway source (e.g., KEGG) is preferred.**

**Response:** We thank the reviewer for this valuable suggestion.

We selected GeneCards as our primary source due to its distinct advantages for exploratory studies. Unlike pathway-specific databases that focus on core components, GeneCards integrates data from nearly 200 authoritative sources (including NCBI, UniProt, and Ensembl), providing comprehensive coverage of genes associated with biological processes. This approach allows identification of not only core pathway members but also upstream regulators, downstream effectors, and modulators that might be missed when using a single curated pathway.

Importantly, following the reviewer's suggestion, we have now performed a complementary analysis to rigorously cross-validate our findings. We intersected the necroptosis gene sets from both GeneCards ( only those genes with a relevance score greater than or equal to twice the median value) and the KEGG pathway (hsa04217), which yielded 44 shared high-confidence genes. The subsequent intersection of these 44 genes with our DEGs consistently identified the same single gene: BIRC3 **(Figure 3 in this document)**.

This additional analysis confirms that our core finding is robust and consistent, regardless of the primary database used. The convergence of evidence from both a comprehensive database (GeneCards) and a curated pathway source (KEGG) strongly reinforces the central role of BIRC3 in the context of PE-related necroptosis.

**Figure.3 Screening of necroptosis-related DEGs (NRDEGs):** The Venn diagram illustrates the intersection and unique genes among three gene sets relevant to the study of necroptosis: GeneCards, KEGG, and DEGs (Differentially Expressed Genes).

**Comment 3: The reported AUC = 0.933 for BIRC3 is based on a very small cohort (n=11). Such results are prone to overfitting and may not reflect true predictive value. The claim of “exceptional diagnostic potential” should be tempered and described as preliminary until validated in independent cohorts**

**Response:** **We sincerely thank the reviewer for raising this critical point. We fully acknowledge that the high AUC value of 0.933 observed in our discovery cohort (GSE66273) is likely optimistic and susceptible to overfitting due to the limited sample size.**

**To directly address this concern and assess the generalizability of BIRC3's diagnostic potential, we performed validation in an independent, external cohort (GSE44711). We are pleased to report that in this validation set, BIRC3 maintained significant diagnostic performance with an AUC of 0.844 (Page 16, line 312-321). While the AUC in the validation cohort is slightly lower—as is typically expected when moving from a discovery set to an independent validation set—it remains a strong indicator of discriminatory power. This successful external validation demonstrates that the diagnostic signal of BIRC3 is genuine, robust, and not merely an artifact of overfitting to our initial small dataset. In the revised manuscript, we have:**

(1) **Tempered our language regarding the initial AUC, describing it as "promising yet preliminary" in the Results section (Page 16, line 319-321).**
(2) **Explicitly discussed the risk of overfitting from the small discovery cohort and the critical importance of the external validation in the Discussion section (Page 25-26).**
(3) **Incorporated the results from the GSE44711 validation cohort, including the AUC of 0.844, into the Results section (Page 16, line 319-321) and the manuscript Fig.5I.**

**Comment 4: Docking scores alone are not sufficient to infer therapeutic relevance. Binding affinity does not necessarily confirm biological activity, specificity, or safety—particularly in pregnancy. The docking results should be framed as hypothesis-generating only, without implying clinical readiness of Withanolide D, Baicalin, or Mangostin.**

**Response:** We thank the reviewer for these critical comments regarding the drug prediction and molecular docking analyses. We agree that these components are exploratory in nature and have revised the manuscript to better reflect this, while also providing the missing methodological details to enhance reproducibility and scientific rigor.

(1) We have removed all language implying therapeutic potential or clinical readiness for Withanolide D, Baicalin, and Mangostin throughout the manuscript, including in the Results and Discussion.
(2) We have reframed the purpose and interpretation of the docking analysis. In the Results section **(Page 21, line 422-423 and line 429-432)**, we now clearly state that these results represent in silico predictions that serve exclusively as a hypothesis-generating platform for future research. We explicitly note that binding affinity does not confirm biological activity, specificity, or safety.
(3) We have repositioned these findings as a starting point for future work, suggesting that the computational predictions should be followed by experimental validation (e.g., in vitro binding assays, functional studies in relevant cell models) before any conclusions regarding therapeutic potential can be drawn **(Page 25, line 494-503).**.

**Comment 5: Both ssGSEA and xCell were applied, but the rationale for using two methods and how differences were reconciled is not clearly explained. Please clarify why both were used and provide a stronger justification for interpreting correlations between BIRC3 expression and immune cell scores.**

**Response:** We thank the reviewer for this insightful comment regarding the rationale for our immune infiltration analysis. We have now clarified these points in the revised manuscript as follows:

(1) Enhanced Multi-Algorithm Strategy and Rationale: We have strengthened our analytical approach by employing not just two, but three complementary deconvolution algorithms: ssGSEA, xCell, and now additionally CIBERSORTx **(Figure 1 in this document).** We have clarified in the manuscript that this multi-algorithm strategy is based on the established principle that different computational methods utilize distinct gene signatures and mathematical approaches. The convergence of findings across multiple methods provides the most robust evidence, allowing us to distinguish algorithm-specific artifacts from biologically consistent signals. The rationale for this approach is now explicitly stated.
(2) Performance of Sensitivity Analysis: As requested, we have now performed a sensitivity analysis using CIBERSORTx. The results of this analysis are included in the new **Supplementary Figure S1** and discussed in the main text **(Page 17-18, line 344-350)**. This analysis confirms that while absolute estimates for specific cell subsets can vary between algorithms—highlighting the inherent limitations of applying these tools to placental tissue—a consistent trend of broad immune dysregulation in PE is observed across all three methods.
(3) Focus on Consensus Findings and Acknowledgment of Limitations: We have reframed our interpretation to focus on the high-confidence, consensus findings. The core conclusion of our analysis is no longer the precise quantification of any single cell type, but the demonstration of a generalized imbalance and dysfunction of the placental immune microenvironment in PE, a finding robustly supported by all algorithms. We explicitly acknowledge the variability between tools and the challenges of placental tissue deconvolution in the revised text **(Page 25, line 508-511)**.
(4) Stronger Justification for BIRC3-Immune Cell Correlations: We have refined our interpretation of the correlations between BIRC3 and immune cell scores. We now emphasize that these correlations represent coordinated patterns of gene expression at the bulk tissue level rather than direct cellular interactions. The consistent positive correlation between BIRC3 and specific immune cells (e.g., T cells) across analytical methods, coupled with its specific expression in these cell types as revealed by our single-cell RNA-seq analysis, provides a multimodal line of evidence suggesting BIRC3's involvement in the immune response within the PE placental microenvironment.

**Comment 6: scRNA-seq analysis is descriptive; statistical comparisons of BIRC3 expression are needed.**

**Response:** We thank the reviewer for this valuable suggestion to strengthen the statistical rigor of our single-cell RNA-seq analysis. In direct response, we have now performed additional quantitative analyses to statistically compare BIRC3 expression between PE and control samples. Specifically, we have:

(1) Increased the analytical sample size for our single-cell analysis by including data from 2 PE and 2 control placentas from the GSE173193 dataset.
(2) Performed a statistical comparison of BIRC3-positive cell proportions between the PE and control groups. Using a Chi-square test, we found that the proportion of BIRC3-positive cells was significantly higher in the PE group compared to the control group (p < 0.05). This analysis provides a population-level, statistically supported insight into BIRC3 dysregulation in PE **(Page 19, line 383-384).**
(3) Complemented this analysis with cell-type-specific resolution. Our analysis revealed that BIRC3 is particularly highly expressed in NKT cells. This allows for a more nuanced understanding of its cell-type-specific involvement (**Figure 4 J-K in this document**).

These new analyses have been incorporated into the revised Results section **(Page 18-19, line 368-387)** and are visualized in a new Figure.

**Figure.4 Single-cell expression analysis and subcellular localization of BIRC3 in tissues and cells.** (A) BIRC3 protein expression overview. (B) The immunohistochemical data of BIRC3 in the placenta were analyzed via the HPA database. (C) BIRC3 subcellular localization. (D) The expression of BIRC3 in the single-cell clusters identified in the placenta visualized by a UMAP plot (HPA database). Each dot corresponds to a cell, and each color corresponds to a cell type. (E-G) UMAP representation of the different 10 cell clusters (GSE173193). (H) The distribution of cell proportions in different groups. (I) Expression map of BIRC3 in different groups.

**Comment 7: Several figures appear blurred and should be provided in high resolution.**

**Response:** We apologize for the issue. We have regenerated all figures (Figures 1-8) in high resolution (minimum 300 DPI) and ensured their clarity upon submission.

**Additional Changes:**

We have thoroughly proofread the manuscript to correct any typographical or grammatical errors and have verified that all in-text citations correspond to the reference list. We believe that addressing the reviewers' comments has significantly enhanced the quality of the manuscript. We would like to express our sincere appreciation to the editors and reviewers for their invaluable feedback and hope that the revised manuscript now meets the standards for publication in PLOS ONE.

Sincerely,

Guili Shi

Department of Obstetrics, Quanzhou Women and Children's Hospital, Quanzhou, China

secret183@163.com
